# Identification of Long Noncoding RNA APOC1P1 as an Oncogene in Clear Cell Renal Cell Carcinoma

**DOI:** 10.1155/2019/2814058

**Published:** 2019-12-01

**Authors:** Chong Sun, Zhonghan Zhou, Haoqing Shi, Fangzhou Li, Guiming Zhang

**Affiliations:** ^1^Department of Spinal Surgery, The Affiliated Hospital of Qingdao University, Qingdao, China; ^2^Department of Urology, The Affiliated Hospital of Qingdao University, Qingdao, China

## Abstract

Renal cell carcinoma (RCC) is one of the most common genitourinary cancers worldwide. Previous evidence shows that long noncoding RNA (LncRNA) APOC1P1 plays an important role in cancer development. However, the role of LncRNA APOC1P1 in ccRCC remains to be explored. LncRNA APOC1P1 expression in 283 ccRCC tissues and 30 normal kidney tissues was detected by quantitative real-time PCR, and its prognostic association with ccRCC was assessed by the Kaplan-Meier method and Cox proportional hazard model. Cell proliferation, apoptosis, migration, and invasion were determined in RCC cells with downregulation of LncRNA APOC1P1 expression. LncRNA APOC1P1 expression was increased in ccRCC tissues compared with normal kidney tissues (*P* < 0.001). Its expression was higher in the Fuhrman grade III and IV group than in the Fuhrman grade I and II group (*P* < 0.05) and significantly upregulated in the advanced stage group (*P* < 0.05). Kaplan-Meier analyses revealed that elevated LncRNA APOC1P1 expression was significantly associated with poor overall survival (*P* < 0.05) but may not be an independent prognostic factor. Knockdown of LncRNA APOC1P1 inhibited cell proliferation, induced apoptosis, and arrested cells at the G1/S phase (*P* < 0.05). Silencing of LncRNA APOC1P1 also led to decreased cell migration and invasion (*P* < 0.05). LncRNA APOC1P1 acts as an oncogene, plays an important role in ccRCC development, and can be considered a prognostic biomarker and therapeutic target in ccRCC patients.

## 1. Introduction

Renal cell carcinoma (RCC) is the most lethal of the common urological cancers and was responsible for 2.2% of newly diagnosed cancers and 1.8% of deaths in 2018 [1, 2]. Historically, clear cell RCC (ccRCC) is the most common subtype and accounts for 80–90% of all RCC cases [3, 4]. Despite the striking progress in cancer detection and treatment, the overall survival (OS) rate in RCC still remains poor [5, 6]. Approximately 1/3 of RCC patients have evidence of metastases at presentation, and the 5-year age-standardized relative survival rate for stage IV disease is 6% compared with 84% for stage I disease [7]. Thus, it is essential to clarify the mechanisms for RCC carcinogenesis and to identify novel biomarkers that can predict its prognosis.

Long noncoding RNAs (LncRNAs) comprise a class of noncoding RNAs that are 200 to 100,000 nucleotides in length without protein-coding capacity [8, 9]. Studies have revealed that LncRNAs play an important role in tumorigenesis and metastasis [10–12]. LncRNA apolipoprotein C-1 pseudogene 1 (LncRNA APOC1P1), which is located at 19q13.2 between APOC-I and APOC-IV, was shown to inhibit apoptosis by decreasing *α*-tubulin acetylation in breast cancer [13]. However, the role of LncRNA APOC1P1 in ccRCC is yet to be explored. In this study, we aimed to determine the expression of LncRNA APOC1P1 in ccRCC and its impact on patients' survival. As well, the biological functions of LncRNA APOC1P1 in RCC cells were also investigated.

## 2. Materials and Methods

### 2.1. Patients and Specimens

A total of 283 ccRCC specimens and 30 adjacent normal renal tissues were collected from patients who underwent radical or partial nephrectomy between January 2007 and December 2010 at the Department of Urology, The Affiliated Hospital of Qingdao University. All specimens were confirmed by postoperative pathological analysis. Tumor staging and grading were confirmed according to the AJCC TNM 2010 classification system and WHO/ISUP 2004 system, respectively. None of the patients received chemotherapy, radiotherapy, or targeted therapy before surgery. Tissues were stored at −80°C immediately after surgical removal for later analysis. Patients were regularly followed up in the clinic or by telephone until June 2018 or date of death. The study protocol was approved by the Ethics Committee of The Affiliated Hospital of Qingdao University, and written informed consent was obtained from all patients before surgery.

### 2.2. RNA Extraction and Quantitative Real-Time PCR (qRT-PCR)

The RNA extraction was performed right before the analyses, and RNA quality control was performed by the Thermo Scientific NanoDrop™ 1000 Spectrophotometer. TRIzol reagent (Thermo Fisher Scientific Inc., Waltham, MA, USA) was used to extract total RNA from tissues or cells according to the manufacturer's protocols and subjected to reverse transcription using a RevertAid First Strand cDNA Synthesis Kit (Thermo Fisher Scientific Inc.). Then, qRT-PCR was performed using a Power SYBR Green PCR Master Mix (Thermo Fisher Scientific Inc.) and an ABI 7900HT Fast Real-Time PCR system (Applied Biosystems, Foster City, CA, USA). Relative expression values of LncRNA APOC1P1 were normalized to *β*-actin. The gene-specific primers sequences were as follows: LncRNA APOC1P1, forward, 5′-GGTCCTGGTGGTGGTTCTGTC-3′, reverse, 5′-CTCCTTCACTTTCCGAAATGTCTC-3′; *β*-actin, forward, 5′-ACCGAGCGCGGCTACAG-3′, reverse, 5′-CTTAATGTCACGCACGATTTCC-3′. All experiments were carried out in triplicate.

### 2.3. Cell Culture and siRNA Transfection

Human RCC cell lines (A498, 786-O, ACHN, and Caki-1) and an immortalized normal human renal tubular epithelial cell line (HK-2) were purchased from The Institute of Cell Research of the Chinese Academy of Sciences (Shanghai, China). A498, 786-O, and HK-2 cells were cultured in RPMI-460 medium (Hyclone; GE Healthcare Life Science, Logan, UT, USA). ACHN and Caki-1 cells were grown in MEM medium and McCoy's 5A medium (Gibco; Thermo Fisher Scientific Inc.), respectively. All media were supplemented with 10% fetal bovine serum (FBS; Hyclone; GE Healthcare Life Science). Cells were cultured at 37°C with 5% CO_2_. Three small interfering RNAs (siRNAs) targeting LncRNA APOC1P1 (siRNA1, siRNA2, and siRNA3) were designed by Guangzhou RiboBio Co. Ltd. (Guangzhou, China). siRNA transfection was conducted with a Lipofectamine 2000 Reagent (Invitrogen, Carlsbad, CA, USA) in accordance with the manufacturer's protocols. The efficiency of knockdown was tested 72 h after transfection.

### 2.4. Cell Proliferation, Cell Cycle, and Apoptosis Assays

Cell proliferation capacity was determined using a Cell Count Kit-8 (CCK-8 assay; Dojindo, Shanghai, China). Briefly, cells were plated in 96-well culture plates at a density of 1 × 10^4^ cells/well and cultured in fresh medium mixed with CCK-8 at a ratio of 10 : 1 for 2 h. The cell density was detected with an enzyme-linked immunosorbent assay microplate reader at 490 nm.

Cell cycle assays were performed using flow cytometry. Briefly, cells were fixed with 75% cold ethanol overnight and then washed with phosphate-buffered saline. Propidium iodide (50 *μ*g/ml) mixed with RNase was added for DNA staining. Then, cells were analyzed by flow cytometry (Beckman Coulter, Brea, CA, USA).

Cell apoptosis was determined by flow cytometry, too. Briefly, after DNA staining with 5 *μ*l of FITC-Annexin V and 5 *μ*l of propidium iodide (BD Biosciences, Franklin Lakes, NJ, USA), cells were incubated at room temperature in the dark for 15 min. An additional 400 *μ*l of binding buffer was then added before analysis by flow cytometry.

### 2.5. Wound Healing and Transwell Cell Invasion Assays

The in vitro wound healing assays were conducted to detect the cell migration capacity. Briefly, cells were seeded in 6-well plates to form a confluent monolayer. An artificial homogeneous scratch wound was made using a sterile 200 *μ*l pipette tip. After incubation for 0, 24, and 72 h, images of the cells were randomly captured under an inverted microscope (IX51; Olympus Corporation Co. Ltd., Tokyo, Japan) at ×100 magnification. Migratory capacity was measured as relative migratory distance. All experiments were carried out in triplicate.

Transwell assays were performed to determine the cell invasion capacity using 24-well Transwell chambers (Corning, NY, USA, No.353097, 8 *μ*m). First, the upper chambers were precoated with 60 *μ*l of Matrigel (BD Biosciences, Franklin Lakes, NJ, USA). Cells were then seeded in the upper chambers in serum-free medium at a density of 4 × 10^4^ cells/chamber, while medium supplemented with 10% FBS was added to the lower chambers. After 48 h of incubation at 37°C, inserts were stained with 0.5% crystal violet (Shanghai Macklin Reagent Co. Ltd., Shanghai, China) for 30 min. The numbers of invaded cells were counted in randomly selected fields under a microscope. Invasion capacity was measured as relative cell counts. All experiments were carried out in triplicate.

### 2.6. Statistical Analysis

SPSS 19.0 software (IBM Corporation, Armonk, NY, USA) was used for statistical analyses. Continuous variables were expressed as mean ± standard deviation (SD). Student's *t*-test and the chi-square test were performed for continuous and categorical variables, respectively. The receiver operating characteristic (ROC) curve was applied to calculate the area under the ROC curve (AUC) for evaluating the diagnostic efficacy of LncRNA APOC1P1. The Kaplan-Meier method was used to detect the influence of LncRNA expression on survival. Univariate and multivariate analyses were carried out by the Cox proportional hazard model. Values of *P* < 0.05 were considered statistically significant.

## 3. Results

### 3.1. LncRNA APOC1P1 Expression in ccRCC Tissues and Normal Renal Tissues

**Figure 1:**
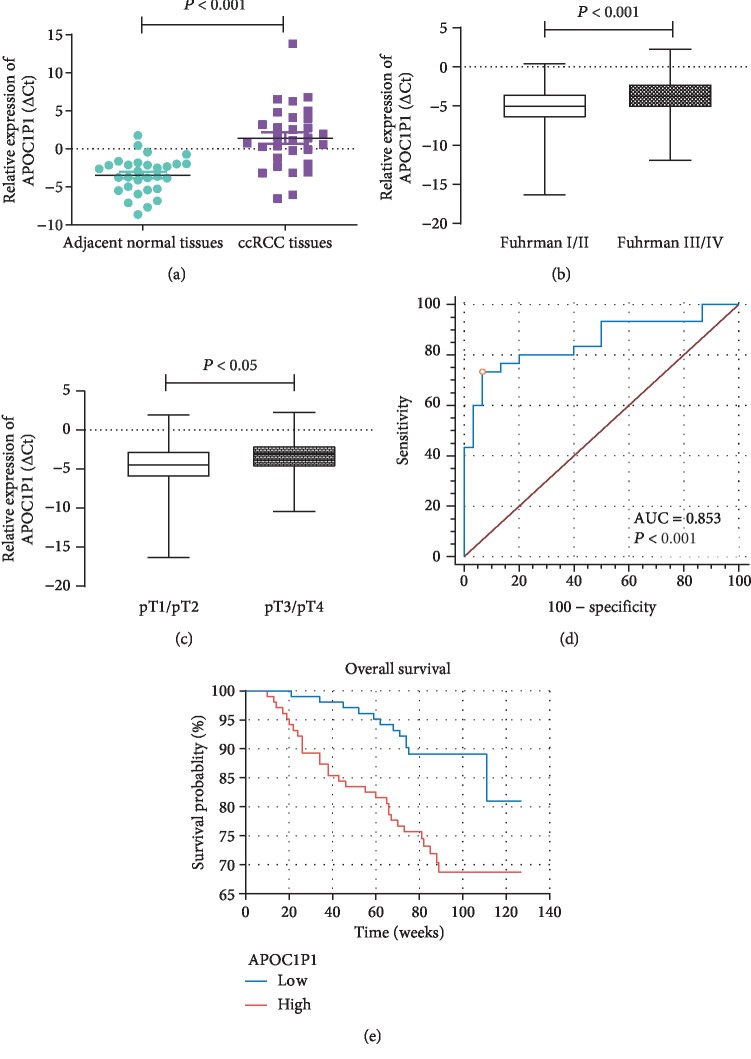
LncRNA APOC1P1 expression in ccRCC tissues and normal renal tissues and influence on overall survival. (a) LncRNA APOC1P1 expression in ccRCC tissues and adjacent normal renal tissues. (b) Comparison of LncRNA APOC1P1 expression with Fuhrman grade patterns. (c) Comparison of LncRNA APOC1P1 expression with stage patterns. (d) Assessment of the diagnostic efficacy of LncRNA APOC1P1 in ccRCC patients. (e) Influence of LncRNA APOC1P1 expression on overall survival. LncRNA APOC1P1 levels were quantified and normalized to *β*-actin. All experiments were carried out in triplicate. The data were presented by *x* ± *s*.

The clinicopathological features of the patients are presented in Table 1. Patients with ccRCC were divided into low (*n* = 142) and high (*n* = 141) expression groups based on the median value (0.40) of relative LncRNA APOC1P1 expression, and clinicopathologic features were compared between the two groups. As summarized in Table 1, increased LncRNA APOC1P1 expression significantly correlated with the Fuhrman grade, T-stage, and Eastern Cooperative Oncology Group (ECOG) grade (*P* < 0.05). However, there was no significant correlation between LncRNA APOC1P1 expression and other clinicopathologic features such as age, gender, BMI, smoking and drinking status, history of hypertension, and diabetes (*P* > 0.05). LncRNA APOC1P1 expression was increased in ccRCC tissues compared with adjacent normal renal tissues (*P* < 0.001, Figure 1(a)). Furthermore, its expression was higher in the Fuhrman grade III–IV group than in the Fuhrman grade I–II group (*P* < 0.05, Figure 1(b)) and significantly upregulated in the advanced ccRCC group compared with the localized ccRCC group (*P* < 0.05, Figure 1(c)). These findings suggest that LncRNA APOC1P1 may play a tumor-promoting role in ccRCC.

The diagnostic efficacy of LncRNA APOC1P1 was evaluated. The ROC curve analysis revealed that AUC was 0.853 (95%CI = 0.738 to 0.931, *P* < 0.001, Figure 1(d)). When the cutoff value = ‐0.442, the diagnostic sensitivity (73.3%) and specificity (93.3%) reached their peak value, indicating that LncRNA APOC1P1 has clinical significance for the diagnosis of ccRCC.

### 3.2. Influence of LncRNA APOC1P1 Expression on Survival

**Figure 2:**
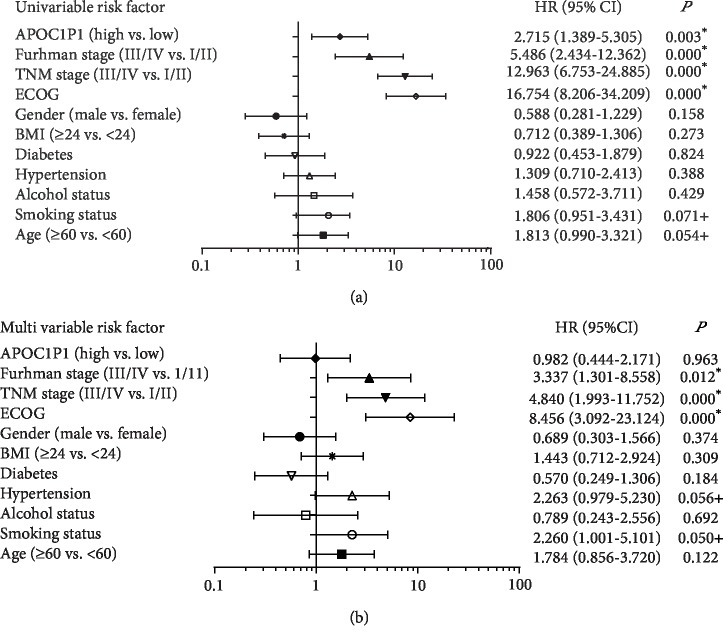
Univariate (a) and multivariate (b) analyses of LncRNA APOC1P1 in ccRCC patients (^∗^*P* < 0.05; ^+^*P* < 0.10).

The follow-up period for the ccRCC patients ranged from 10 to 127 months (mean: 82.2 months). Kaplan-Meier analysis revealed that elevated LncRNA APOC1P1 expression was significantly associated with poor OS (*P* < 0.05, Figure 1(e)). In the univariate Cox regression, Fuhrman stage, TNM stage, Eastern Cooperative Oncology Group (ECOG) grade, and LncRNA APOC1P1 expression were prognostic factors in ccRCC patients (Figure 2(a)). However, the multivariate Cox regression indicated that LncRNA APOC1P1 expression may not be an independent prognostic factor (Figure 2(b)).

### 3.3. Effect of LncRNA APOC1P1 Knockdown on Proliferation, Apoptosis, and Cell Cycle

**Figure 3:**
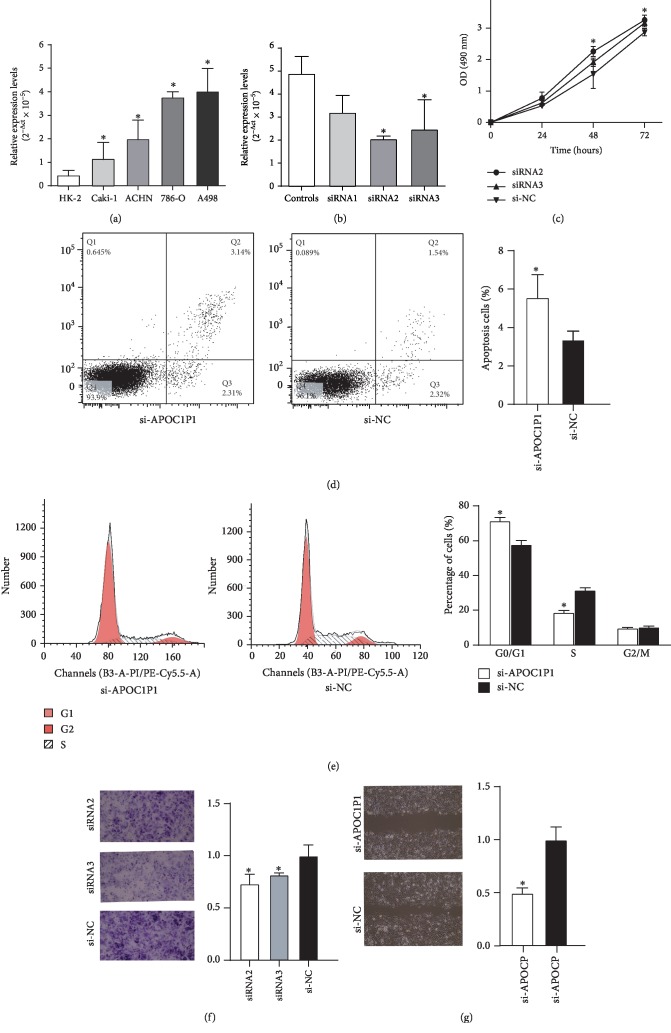
Effect of LncRNA APOC1P1 knockdown on proliferation, apoptosis, cell cycle, cell invasion, and migration capacity in ccRCC cells. (a) Expression of LncRNA APOC1P1 in human ccRCC cell lines (A498, 786-O, ACHN, and Caki-1) and an immortalized normal human renal tubular epithelial cell line (HK-2). (b) Validation of cell transfection efficiency of three siRNAs in A498 cells. (c) Cell proliferation measured by CCK-8 assays. (d, e) Cell apoptosis rate (d) and cell cycle (e) measured by flow cytometry. (f, g) Effect of LncRNA APOC1P1 knockdown on cell invasion and migration measured by Transwell chamber assays (f) and wound healing assays (g) (^∗^*P* < 0.05).

We measured the expression of LncRNA APOC1P1 in four human ccRCC cell lines (A498, 786-O, ACHN, and Caki-1) and an immortalized normal human renal tubular epithelial cell line (HK-2) by qRT-PCR. As shown in Figure 3(a), there was higher expression of LncRNA APOC1P1 in all four ccRCC cell lines compared with HK-2 cells (*P* < 0.05), consistent with the expression pattern of LncRNA APOC1P1 in the tissue specimens. A498 cells exhibited the highest LncRNA APOC1P1 expression (*P* < 0.05), and thus, the A498 cell line was selected for further analyses.

Transfection with LncRNA APOC1P1 siRNAs was performed to downregulate the expression of LncRNA APOC1P1, and transfection with siRNA2 and siRNA3 successfully decreased LncRNA APOC1P1 levels in A498 cells compared with the positive control group (*P* < 0.05, Figure 3(b)). Moreover, siRNA2 exhibited the best transfection efficiency. Therefore, knockdown of LncRNA APOC1P1 was performed with siRNA2 to investigate its biological function.

Cell proliferation was evaluated by CCK-8 assays after transfection with si-LncRNA APOC1P1 (in both the siRNA2 and siRNA3 groups). The results indicated that si-LncRNA transfection led to cell growth arrest in A498 cells compared with the si-NC group (*P* < 0.05, Figure 3(c)).

Apoptosis and cell cycle analyses were performed at 48 h after siRNA transfection into A498 cells. As shown in Figure 3(d), the apoptosis rate was clearly increased after LncRNA APOC1P1 downregulation compared with the si-NC transfection group (*P* < 0.05). In the cell cycle analyses, the proportion of cells in the G1-phase was significantly increased after transfection, while the proportion of cells in the S-phase was notably lower than that in the control group, suggesting that LncRNA APOC1P1 knockdown induced G1/S arrest (*P* < 0.05; Figure 3(e)).

### 3.4. Effect of LncRNA APOC1P1 Knockdown on Cell Invasion and Migration

Wound healing and Transwell chamber assays were conducted to clarify the effect of LncRNA APOC1P1 on cell migration and invasion. Compared with the si-NC group, cell migration was significantly inhibited after si-LncRNA APOC1P1 transfection, and cell invasive capacity was also markedly reduced (*P* < 0.05, Figures 3(f) and 3(g)). Compared with the si-NC group, cell invasive capacity was reduced after both siRNA2 and siRNA3 transfection, indicating that the off-target effect can be excluded (*P* < 0.05, Figure 3(f)). Cell migration was also significantly inhibited after si-LncRNA APOC1P1 transfection (*P* < 0.05, Figure 3(g)).

## 4. Discussion

Over 200,000 new RCC cases are diagnosed every year, and more than 1000,000 related deaths occur worldwide. In 2015, the International Agency for Research on Cancer predicted a 22% increase in the number of people developing RCC by 2020 [14, 15]. Thus, establishment of a sensitive and reliable molecular marker to aid in its diagnosis and treatment is in urgent need. In the last decades, many studies have shown that LncRNAs have multiple functions in a wide range of biological processes, such as the cell proliferation, cell apoptosis, cell cycle arrest, and cell migration and invasion [16]. Plenty of reports have investigated the diagnostic and prognostic value of LncRNAs in various cancers [17, 18].

Apolipoprotein C1 (APOC1), expressed primarily in the liver, encodes a member of the apolipoprotein C1 family which plays a crucial role in lipid metabolism [19–21]. It stimulates cell proliferation and prevents cell apoptosis [22]. *APOC1P1*, located at 19q13.2, is the pseudogene of *APOC1*. Generally, the antisense transcripts produced from pseudogenes can hybridize to corresponding mRNAs, forming dsRNAs cleaved by Dicer to endogenous siRNAs [23]. LncRNA APOC1P1 was found overexpressed in breast cancer, and its upregulation promotes cell proliferation [13]. However, the role of LncRNA APOC1P1 in ccRCC has not been reported. Therefore, the present study focused on the effects of LncRNA APOC1P1 during onset and progression of ccRCC.

It is reported that LncRNA APOC1P1 is overexpressed in breast cancer [13]. Similar results were observed in our study. The expression of LncRNA APOC1P1 was upregulated in ccRCC tissues compared with normal renal tissues. In addition, elevated expression was associated with higher Fuhrman grade and clinical stage. These results suggest that LncRNA APOC1P1 plays a role in tumor occurrence and development, which is inconsistent with previous study [13]. To assess its prognostic value in ccRCC patients, survival analysis was performed, revealing that LncRNA APOC1P1 expression was associated with worse OS. While the univariate Cox regression reached statistical significance, the multivariate Cox regression revealed that its expression may not be an independent prognostic factor. We attribute these findings to the close correlations between LncRNA APOC1P1 expression and clinicopathological factors, such as the Fuhrman stage, TNM stage, and ECOG grade. These conclusions require validation using large-sample, multicenter datasets. Furthermore, functional experiments *in vitro* were conducted to evaluate the biological roles of LncRNA APOC1P1 in ccRCC. Knockdown of LncRNA APOC1P1 expression inhibited cell proliferation, induced apoptosis, and arrested cells at G1/S phase. Silencing of LncRNA APOC1P1 also led to decreased cell migration and invasion. These findings suggest that LncRNA APOC1P1 may impact the clinicopathological features of ccRCC by affecting cell proliferation and invasiveness. A previous study revealed that LncRNA APOC1P1 can bind to tubulin and then increase *α*-tubulin acetylation and inhibit apoptosis [13]. We assume that in ccRCC, LncRNA APOC1P1 might function in the same process. Clearly, further researches about the expression and function of LncRNA APOC1P1 in cancer initiation and progression are warranted.

## 5. Conclusions

In summary, we have identified elevated LncRNA APOC1P1 expression in ccRCC. Our findings indicated that LncRNA APOC1P1 expression was significantly associated with OS, but may not be an independent prognostic factor. LncRNA APOC1P1 plays an important role in the onset and progression of ccRCC by affecting cell proliferation and invasion. As a protooncogene, LncRNA APOC1P1 can be considered a prognostic biomarker and therapeutic target in ccRCC patients.

## Figures and Tables

**Table 1:** Clinicopathological features of patients with ccRCC.

Parameters	Group	Total	LncRNA APOC1P1 expression		*P* value
			Low	High	
Age	<60	180	85	95	0.217
	≥60	103	57	46	
Gender	Male	194	96	98	0.798
	Female	89	46	43	
BMI	<24	128	68	60	0.404
	≥24	155	74	81	
Smoking	No	223	111	112	0.885
	Yes	60	31	29	
Alcohol	No	259	131	128	0.676
	Yes	24	11	13	
Hypertension	No	184	88	96	0.319
	Yes	99	54	45	
Diabetes	No	211	111	100	0.174
	Yes	72	31	41	
Fuhrman grade	I/II	133	50	83	<0.001
	III/IV	150	92	58	
T-stage	T1/T2	255	122	133	0.027
	T3/T4	28	20	8	
ECOG	No	266	127	139	0.002
	Yes	17	15	2	

## Data Availability

The data used to support the findings of this study are included within the article or the supplementary information file(s).
